# Pituitary incidentalomas - How often is too often?

**Published:** 2009

**Authors:** Mara Carsote, Corina Chirita, Anda Dumitrascu, D Hortopan, Simona Fica, Catalina Poiana

**Affiliations:** * Department of Endocrinology, “Carol Davila” Univesity of Medicine and Pharmacy, Bucharest, Romania; ** “C.I.Parhon”, National Institute of Endocrinology, Bucharest, Romania

## Abstract

**Introduction**:

Clinical nonfunctional pituitary microadenomas, also known as incidentalomas are accidental observations made due to the application of high resolution imaging techniques as computed tomography or magnetic resonance. There are no standards regarding the follow-up of these tumors and taking into account their increased frequency among general population (during the last years they were based on the high performances of the imaging methods), we decided to study their dimensions and their evolution over time. We have also analysed their behavior with respect to their endocrine phenotype and the minimum period of time needed for the repetition of the imagistic procedure.

***Aim***:

To observe the natural evolution of tumors’ dimensions in a group of patients, diagnosed with nonfunctional pituitary microadenomas based opon hormonal measurements and computed tomography scan.

**Method**:

There is a retrospective observational study on 149 patients hospitalized in our Clinic between 1994 and 2006. Initially, all the pituitary hormones and the computed tomography were performed. Only nonfunctional microadenomas were included (the maximum diameter 11 mm). 69 patients were examined for a long period of time - 29.75 +/- 24.79 months by CT scan and secretory profile, repeated at different periods of time.

**Results**:

At the end of 29.75 months, the aspect of microadenoma was still present, without any statistically significant changes of the diameter. One of the cases became macroadenoma and another proved to be a microprolactinoma. Only 5 cases of all 149 presented a double lesion. No case of pituitary apoplexy was registered.

These observations lead to the conclusion that it is not necessary to repeat the computed tomography scan sooner than 2 years once the diagnosis of incidentaloma was established.

## Introduction

Improvement in diagnostic imaging techniques over the last several years has led to an increasing recognition of asymptomatic lesions in pituitary area. The analysis based on the latest radiological methods induced the appearance of terms such as “benign anatomical mistakes” or acronyms such as: “VOMIT” for ”victims of modern imaging technology” [**1**,**2**].

A definition of incidentalomas as given in 2002 is the following ”totally asymptomatic nonfunctional tumors, clinically and biochemically silent, incidentally discovered in an asymptomatic patient” [**2**].

Some lesions may increase in size, due to impaired pituitary hormone production, so most of them will not modify their size and will never produce tumoral and hormonal symptoms. 

The type of the initial endocrinological assessment and the required frequency and length of the follow-up for pituitary incidentaloma need to be carefully determined, taking into account the cost effectiveness. So, it is important to get precise information about the natural history of every pituitary lesion [**3**].

A recent metaanalysis found an overall prevalence of pituitary adenomas to be 14.4% in autopsies and 22.5% in radiological studies [**4**]. Others reported the ocurrence of incidental pituitary tumors to be 4-20%, which is consistent compared to the results in necropsy studies [**5**].

The management of incidentalomas is controversial [**6**]. We should consider the problem of pituitary incidentalomas, taking into account the following information:

- They represent common results in asymptomatic individuals.

- The endocrine evaluation is essential, as both hypersecretion and hypopituitarism may occur.

- Spontaneous regression or haemorrhage may appear.

- In the absence of visual field defects and endocrine abnormalities, the patients need no treatment, only adequate follow-up by radiological methods [**7**].

## Subjects and Methods

We made a retrospective observational study. The lot consists of 149 patients, hospitalized in “C.I.Parhon” Institute of Endocrinoly, Bucharest, between 1994 and 2006.

At first, pituitary computed tomography scan was performed, based on clinical results such as persistent cefalalgic syndrome or skull radiologic changes as enlargement of the sella turcica or inhomogeneous aspect at this level.

Only the patients with tumors of maximum 1o mm such as microadenomas (maximum diameter less than 9mm) and mezoadenomas (maximum diameter between 9 and 10 or 11mm) were included. No macroadenoma was initially included. A visual field exam was also obtained. Initially, the pituitary hormones were determined by radioimmunoassay. Inhibition test with dexamethasone (1mg overnight or the standard test - 2mg for 2 days) was practiced in order to rule out subclinical Cushing syndrome in the case of obese or/and type 2 diabetic patients .

Based on the secretor’s profile, we excluded all the cases presenting pathological hormonal pituitary secretion as prolactinoma, acromegaly and Cushing’s disease. No cases of tireotropinomas or gonadotropinomas were found either. 

Out of all 149 patients, 69 were later examined in the same manner (hormonal secretor profile and CT scan); some of them more than once at different periods of time (mean 29 months).

## Results

The sex ratio was 139 females and 10 men. The mean age at diagnosis: 40.87 +/-12.87 years old (minimum 14 and maximum 72 years old).

In the beginning, the hormonal values were in normal parameters as shown in **Table 1**.

**Table 1:** The hormonal profile of the studied patients

Hormone	Mean	Standard deviation	Normal range	Units
Prolactin	4.41	13.53	2.8-29.2	ng/mL
GH	5.65	7.26	0-7	ng/mL
TSH	4.57	9.75	0.5-4.5	µUI/mL
LH	14.08	12.68	8.7-76	mUI/mL
FSH	25.96	33.73	3.4-33.4	mUI/mL
ACTH	28.82	11.21	9-60	pg/mL

The computed tomography dimensions in the beginning were all less than 11mm (**Table 2**).

**Table 2:** Tumors’ dimensions in the study group in the beginning

Diameter (cm)	Mean	Standard deviation	Minimum	Maximum
Transversal	0.58	0.20	0.25	1.1
Longitudinal	0.39	0.12	0.18	0.88

**Fig. 1:**
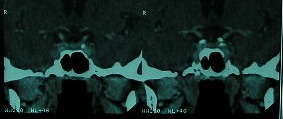
G.A., female patient, age 43, CT pituitary scan: coronal view of bilateral pituitary lesions patients

**Fig. 2:**
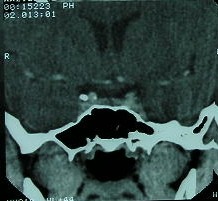
B.I., female patient, age 21, CT pituitary scan: coronal view of a left laterosellar tumor of 0.4 by 0.54 cm

**Fig. 3:**
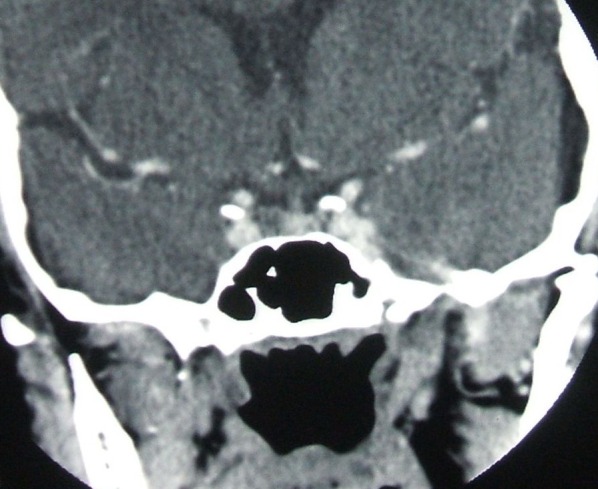
D.V., female patient, age 33, CT pituitary scan: coronal view of a right laterosellar tumor of 0.9 by 0.6 cm

The localization of the tumor was on the right pituitary area in 81 cases (54.36%), on the left side in 42 cases (28.18%), basal in 21 (14.09%) and, only five cases (3.35%) presented bilateral microadenomas (**Fig. 1**, **2**, **3**).

Prolactin at different intervals and only one female was later found with microprolactinoma (prolactinemia over 150 ng/ml, outside the pregnancy or medication/other medical condition known to raise prolactine levels).

Among all the cases, 69 were examined for a long period of time - 29.75 +/- 24.79 months (between 6 and 96 months), by a series of computed tomographies performed at different periods of time. One case turned out to be macroadenoma over several years.

Our final results did not include the macroadenoma, neither the microprolactinoma.6 out of 149 cases actually presented double pituitary lesions such as two microadenomas. In 5 of them there were two microadenomas from the beginning, but in the other patient, the 6th one, we have found a thid pattern 7 months later.

The mean diameters at the end of the registered period also proved aspects of microadenomas (**Table 3**).

**Table 3:** Tumors’ dimensions of the patients at the end of the study

Diameters (cm)	Mean	Standard deviation	Minimum	Maximum
Transversal	0.57	0.17	0.3	1
Longitudinal	0.38	0.09	0.2	0.6

The diameters changed in the 69 patients in the following way: the transversal diameter increased in 25 patients (36.23%), decreased in 31 patients (44.92%) and remained unchanged in 13 patients (18.84%). Regarding the longitudinal diameter, it increased in 19 cases (27.53%), decreased in 34 cases (49.27%) and it was identical in 16 patients (23.18%) (**Table 4**).

**Table 4:** The variation of the tumor dimensions over the study period

Diameter	Increase	Decrease	Unchanged
Transversal	25 patients	31 patients	13 patients
Longitudinal	19 patients	34 patients	16 patients

The difference between the transversal and the longitudinal diameter in the beginning and in the end showed no statistical significance (T test) p values = 0.2634, respectively 0.1307.

## Discussions

Pituitary adenomas arise from adenohypophyseal cells. The true incidence and prevalence of the pituitary adenomas are difficult to be established, but epidemiologic studies suggest a prevalence of about 20 cases per 100 000 population and an incidence of 0.5 to 7.4 new cases per 100 000 population [**8**].

An apparent increase of the incidence of pituitary tumors in the last two decades may be related to the introduction of computed tomography, magnetic resonance imaging (MRI) and a variety of radioimmunoassay techniques for pituitary hormones [**9**].

The differential diagnosis of mass found in the sellar area includes: aneurysms of internal carotid artery, craniopharyngiomas, meningiomas of tuberculum sellae, gliomas of hypothalamus and optic nerves, dysgerminomas, cysts, hamartomas, metastases, sarcoidosis, eosinophilic granulomas, sphenoid sinus mucoceles [**7**].

One of the greatest challenges of modern endocrinology is to distinguish between the vast majority of clinically insignificant changes from other masses requiring further management such as hormone-secreting tumours and malignant lesions [**10**]. There are some experiments regarding endocrine and neurological profile in immunohystochemistry like galanin-3 expression which is ubiquitous in tumors of sellar region but also in the nervous system [**11**]. The cell proliferation markers may be immunostained by using monoclonal K1-67 antibody and monoclonal anti-topoisomerase II alpha antibody. Typically, microincidentalomas are not aggressive [**12**].

Pituitary adenomas are usually benign slow-growing tumors. Those functional are more common in early ages while most of the nonfunctional adenomas are present mostly at older ages. There is a multistep model of pituitary tumor genesis that helps incidentaloma develop only in persons who already have instrinsec pituitary defects. Some theories suggested that if the age related correlation exists, it might be caused by the decline of the sex hormones levels and the raising of the gonadotropes [**13**].

Most of the nonfunctional adenomas reveal secretory granules at immunohistochemistry studies, suggesting a hormonal synthesis, but they fail to secrete functional hormones. Recent studies showed that 30% of incidentalomas are gonadotropinomas, 40% are plurihormonal non-secreting adenomas and the rest of 30% are not reacting to any anterior pituitary hormone antibodies. Pituitary incidentalomas are defined as adenoma discovered by CT or MRI examination in the absence of any symptoms or clinical-results which suggest pituitary-dependent disease. According to some statistics adenomas less then 10mm have been reported in 1.5-27% of pituitary autopsy [**14**].

Usually, autopsy series estimates that 10% of the adult population has these kinds of lesions [**15**].

The prevalence of pituitary incidentalomas found by MRI is 10% [**16**]. Almost 99.5% of them are microadenomas [**9**].

Taking into consideration the fact that the etiology of the pituitary incidentalomas is unknown, some authors tried to see if it is associated with others incidentalomas such as adrenals. In one study, pituitary MRI scan on people with adrenal incidentalomas found normal aspect on 15 patients, empty sella on one and a sellar cyst in another. The co-presence of those two types of incidentalomas is not usually detected [**17**].

Some subclinical hypersecretion syndromes may increase morbidity. For instance, subclinical Cushing’s disease may contribute to poor control of blood sugar and blood pressure levels. Still, 24-hour urinary free cortisol has a very low yield in the absence of clinical features characteristic for Cushing’s disease. Salivary cortisol has higher sensitivity and specificity for Cushing syndrome than the serum test. Now attention is focused on the relationship between subclinical cortisol excess and the metabolic syndrome. Thus, 1 mg dexamethasone suppression test over night should be given in all these cases. Subclinical Cushing syndrome is present in 2% of the overweight patients with poor controlled diabetes [**18**].

The minimum baseline tests also include serum prolactin (some statistics showed that after serial follow-up, 10% of pituitary incidentalomas secrete, most commonly, prolactin) [**19**], IGF-1, or TSH (especially in iodine deficient areas) [**19**,**20**].

Patients with incidentalomas do not need any therapy. The size of the tumor is permanently monitored for a year, increasing the duration after 2 or 3 years [**9**].

Based on a five hundred and six patients study other authors suggested that the surveillance by MRI is necessary in silent pituitary mass every 6 months for the first 2 years and then once a year [**21**].

Similar recommendations are suggested by other authors too: MRI should be performed every 6 months for 1 year, then annually for a year or two. A serum prolactin measurement is the most cost-effective test to be performed [**4**].

There are also studies regarding pituitary nonsecreting adenomas of grade A and a part of those with grade B according to Hardy’s classification. The study shows that surgical treatment in not necessary unless ophthalmologic and endocrinological dysfunction are noted [**22**]. Surgical approach of these patients also proved an increased rate of tumor regrowth of 15-40 % [**23**,**24**].

Data from literature suggest that patients with incidentalomas have a slightly increased risk of morbidity and mortality [**25**].

Correct identification of patients with incidentalomas is important so as to avoid unnecessary pituitary surgery or expensive imagisting surveillance. We should also consider the significant anxiety of the patient because of the fear of future problems, including tumor growth or secretion.

Due to the fact that the natural history of this entity is not clearly known, guidelines on the management of pituitary incidentaloma need to be further established.

## Conclusion

Based on our observations, the computed tomography should not be performed sooner than 2 years, once the adequate diagnosis of incidentaloma is established (this refers to the non-secreting features of the pituitary adenoma and/or its potential growing from morphological and anatomical point of view). So, our answer to the question “how often is too often” is “less than 24 months”.
